# Radiofrequency stimulation of the dorsal root ganglion as a diagnostic tool for radicular pain syndromes: six representative cases

**DOI:** 10.1186/s44158-024-00194-y

**Published:** 2024-09-03

**Authors:** Laura Demartini, David Michael Abbott, Cesare Bonezzi, Silvia Natoli

**Affiliations:** 1 Pain Unit, ICS Maugeri IRCCS, Pavia, Italy; 2 Department of Surgical, Pediatric and Diagnostic Sciences, University of Pavia, 27100 Pavia, PV Italy; 3 Fondazione IRCCS Policlinico San Matteo, 27100 Pavia, PV Italy; 4 Resident of Anesthesia, Intensive Care and Pain Medicine, University of Pavia, Pavia, Italy

**Keywords:** Radiofrequency, Pulsed radiofrequency treatment, Dorsal root ganglia, Diagnosis of neuropathic pain

## Abstract

**Background:**

We discuss the diagnostic benefit of pulsed radiofrequency (PRF) of the dorsal root ganglion (DRG) in a case series of patients with different pathologies. We expand the diagnostic potential of DRG stimulation beyond paresthesia mapping by using DRG stimulation to help determine the role of the DRG in the patient’s pain and narrow down the etiology. In some cases, DRG stimulation was also part of the treatment plan.

**Methods:**

Six patients underwent DRG radiofrequency as a diagnostic/therapeutic step before considering implantation of a DRG neurostimulator. First, patients underwent a basic bedside neurological evaluation. Next, an electrode was placed in the epidural space through the sacral hiatus or between vertebral laminae. Then, sensory stimulation was applied at 50 Hz and gradually increased from 0.1 V until the patient reported paresthesia or until a maximum intensity of 2 V was reached. Patients were asked to describe where the stimulation was felt and outline the anatomical area the paresthesia covered. Then a motor stimulation was applied at 2 Hz until muscle twitching was reported by the patient or observed by the physician.

**Results:**

The information obtained helped diagnose the type of lesion as principally preganglionic, ganglionic, or postganglionic. This information guided patient management.

**Conclusion:**

PRF of the DRG can provide valuable diagnostic information and is a useful step before ganglionic electrode implantation. In all cases, PRF of the DRG provided valuable diagnostic information and guided management options.

## Background

Pulsed radiofrequency (PRF) applied to the dorsal root ganglia (DRG) is a therapeutic neuromodulation technique effective in various types of chronic neuropathic pain syndromes [1, 2]. PRF is a safe and effective way to target specific sensitive fibers or ganglia and provide neuromodulation to the fibers at the source of a given neuropathic pain. PRF has been shown to be especially effective for cervical or lumbosacral radicular pain, postherpetic neuralgia, and occipital neuralgia [3]. The DRG is an appealing target for PRF as structural and molecular changes to the DRG may be the source of chronic pain [4, 5]. In addition, because of the DRG’s location between the dorsal column of the spinal cord and the peripheral spinal nerve, PRF of the DRG can help diagnose the source of radicular pain.

Dermatome maps are often unreliable indicators of the spinal level from which neuropathic pain originates, as they do not account for the pain of deeper anatomical structures such as muscles and bones, nor do they account for functional changes of the ganglia [6]. For example, Chapman et al. discussed how the T12 DRG seems to be especially involved in low back pain and stimulation of this ganglia provides significant pain relief, even though dermatome models show the lower back to be innervated by levels L1-L5 [7].

Most peripheral nerves are formed by several roots and some areas of the body receive sensory innervation from more than one nerve [2]; this convergence of first-order neurons onto second-order ascending neurons is the proposed mechanism behind somatic referred pain [6]. PRF of the DRG at a given level creates a peripheral paresthesia in a specific area that may or may not cover the area of the patient’s pain. After mapping different DRG levels, it is then possible to apply PRF or implant electrodes that will cover the area(s) most involved in the patient’s pain [8, 9].

In 2014, Zuidema et al. demonstrated this as they used paresthesia mapping of a given painful area with PRF to selectively identify the best target(s) for DRG electrode placement in patients with refractory groin pain [8].

Hunter et al. presented a case series of patients with post-amputation pain in the lower limbs in which selective RF stimulation of different ganglia was useful to identify the best targets for DRG stimulation to cover areas of complex pain [9].

Although these studies are compelling, the number of studies demonstrating the benefits of PRF of the DRG in localizing and treating radicular pain is lacking.

Our work adds to the literature by discussing the diagnostic benefit of DRG stimulation in a small group of patients with different pathologies. We expand the diagnostic potential of DRG stimulation beyond paresthesia mapping by using DRG stimulation to help determine the role of the DRG in the patient’s pain and narrow down the etiology. Therefore, in patients with relatively localized neuropathic pain that was unresponsive to pharmacologic therapy, we used DRG stimulation to provide pain relief and obtain diagnostic information that helped us plan future interventions. Indeed, in some patients, the information obtained through the PRF guided further management by helping us understand the best DRG to treat (or not treat) and the optimal spinal cord stimulation (SCS) algorithm settings.

## Models of peripheral neuropathic pain

Neuropathic pain is present in different diseases affecting the peripheral nervous system from roots to nerve endings, but patients with the same disease (e.g., diabetic polyneuropathy or radiculopathy) can present with no symptoms or with mild to severe pain. To improve outcomes of pharmacotherapy, attempts have been made to understand the pathophysiological mechanisms underlying these symptoms [10].

Baron et al., in 2017, described three clusters of patients with neuropathic pain, based on sensory profiling with quantitative sensory testing (QST) along with the corresponding pathophysiological mechanisms [11].

- *Sensory loss*: loss of small and large fiber function with the possible presence of paradoxical heat sensation. Spontaneous pain may occur and is likely due to ectopic action potentials generated proximal to injured nociceptors. These proximal sites include dorsal root ganglion or deafferented central nociceptive neurons.
- *Thermal hyperalgesia*: large and small sensory fiber function is relatively preserved, and patients present with hot and cold hyperalgesia. Low-intensity dynamical mechanical allodynia (DMA) (where gentle brushing of the skin provokes pain) is one characteristic of thermal hyperalgesia. Ongoing hyperactivity in surviving nociceptors may be responsible for ongoing pain and may lead to some central sensitization in the dorsal horn.
- *Mechanical hyperalgesia*: predominant loss of cold and heat-sensitive small fiber function in combination with blunt pressure hyperalgesia and pinprick hyperalgesia. This cluster of neuropathic pain may be associated with intense and frequent DMA. Central sensitization is prominent for mechanical stimuli. Ongoing pain in this subgroup indicates spontaneous activity in the nociceptive system, which may originate in the peripheral and/or central nervous system.

Better understanding of the pathophysiology behind neuropathic pain can guide therapeutic options. These phenotypes are interesting but do not identify the location of a lesion along a primary or secondary neuron and therefore do not always help with treatment planning. Unfortunately, the drugs available to treat neuropathic pain still lack evidence of consistent favorable outcomes and the complex mechanisms of action make it difficult to apply drug treatment based on Baron’s clusters [12]. To further complicate the picture, no study has convincingly demonstrated the efficacy of invasive treatments for neuropathic pain. These problems are partially a result of an incomplete pathophysiological understanding; the mechanisms contributing to the three sensory phenotypes are hypothesized, but not completely demonstrated [13]. Among such diagnostic and therapeutic challenges, PRF can help shed light on the role of DRG in the patient’s neuropathic pain and help guide decision-making in terms of treatment options.

## Radiculopathy/plexus lesions

Radicular lesions (radiculopathies) are mainly due to anatomic compression (such as disk herniation and spinal stenosis) but can also be caused by metabolic disorders, toxicity, neoplastic diseases, radiotherapy, trauma, and radicular cysts [14]. Pain is evoked by ectopic discharges from a dorsal root or its ganglion. Inflammatory processes can increase nerve root sensitivity, and, in such cases, mechanical stimulation can evoke radicular pain. If no inflammatory-mediated sensitization of the nerve root takes place, mechanical traction or compression of the nerve root does not cause pain [15]. Animal studies indicate that in contrast to the dorsal root, compression of the dorsal root ganglion does cause pain [6]. Compressing an inflamed dorsal root or a non-inflamed dorsal root ganglion causes Aβ, Aδ, and C fibers to discharge, which may explain the unpleasant electrical character of radicular pain that is different from nociceptive pain [6].

**Fig. 1:**
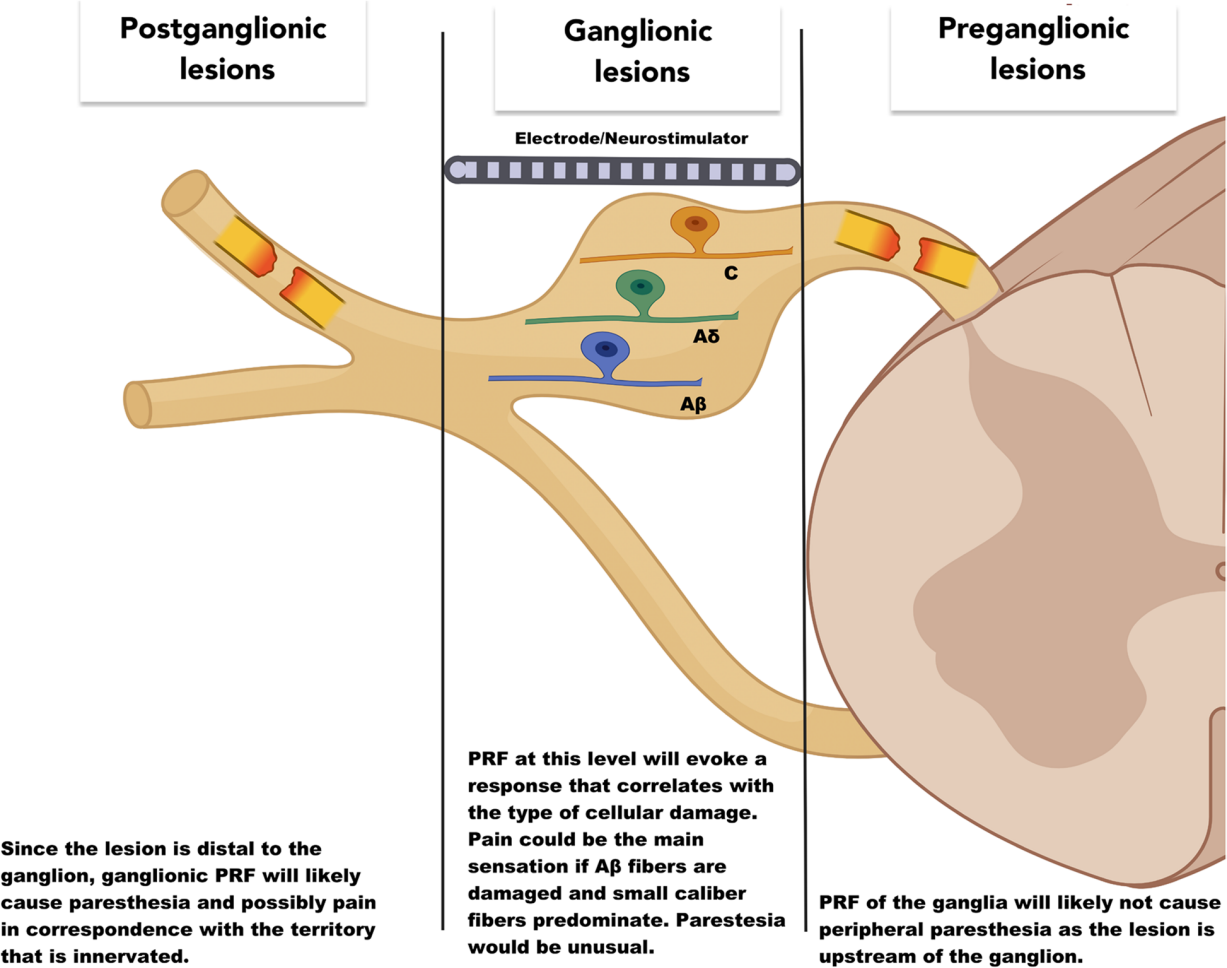
Representation of the DRG with the soma of the pseudo-unipolar neurons that reside within it. DRG stimulation with an electrode/neurostimulator is depicted above the nerve. Lesions are shown in their corresponding site. Created with BioRender.com

The site of the lesion, proximal or distal to the DRG, is related to different pathophysiological mechanisms. Damage to a peripheral neuron (postganglionic), the DRG itself, or to the nerve root proximal to the DRG (preganglionic) creates a painful sensation due to ectopic impulses (Fig. 1).

These ectopic impulses may originate at the site of the lesion or proximal to the lesion. For example, ectopic impulses originate in the DRG when the lesion is postganglionic or when the DRG is only partially damaged. However, ectopic discharges originate in second-order neurons when lesions are preganglionic such as in brachial plexus avulsion. Sometimes the location of the lesion can be inferred with the help of neurologic signs, such as Horner’s syndrome in the case of brachial plexus avulsion, but stimulation of the DRG helps determine if the ectopic impulses originate in the DRG or proximal to the DRG. Knowledge about DRG functionality helps determine the best treatment strategy.

According to Baron et al., radiculopathy is mainly associated with sensory loss and thermal hyperalgesia [11], but, clinically, we cannot define the site of lesion, and sometimes, even neurophysiology cannot give us precise information.

When the lesion is preganglionic for one or more roots, sensory nerve action potentials (SNAPS) produced by electrical stimulation of peripheral nerves may be present even in a completely anesthetic limb [16]. The amplitude of SNAP decreases if there is compression of neural tissue at the level of, or distal to, the DRG with distal axonal degeneration. Recording somatosensory evoked potentials (SEPs) from the sensory cortex or the cervical spinal cord, while stimulating a major nerve trunk of the affected limb increases the diagnostic power [16]. In certain cases, the preganglionic lesion may be partially or completely obscured (as far as electrophysiological recordings are concerned) by coexisting postganglionic damage to the same fibers, so there could be the need of a myelogram to detect a root avulsion even in the presence of a total postganglionic lesion [17]. Rat studies and some human studies suggest MRI can help diagnose root avulsions too, though the concept is new and systematic verification is needed [18, 19]. As both EMG and X-ray findings can be misleading, surgical exploration may be the final approach; even then, the anatomy is not always clear. When the anatomy is unclear during surgical exploration, intraoperative stimulation and recording procedures have been recommended [17]. DRG stimulation may bypass other invasive strategies, save time, and provide diagnostic information that is useful in future treatment planning.

## Postherpetic neuralgia

Varicella-Zoster virus (VZV) is a herpesvirus that remains latent in the DRG or cranial ganglia of infected patients until reactivation, typically when the patient is in an immunosuppressed state. During reactivation, the virus travels along the central and peripheral dendrites until it reaches the skin where it causes shingles, usually involving one or more dermatomes [20]. Postherpetic neuralgia (PHN) is defined by persistent pain despite resolution of shingles. PNH is an important cause of morbidity, and pharmacological treatment is often insufficient in managing the persistent pain [21]. Different studies have demonstrated the correlation between important histological and molecular changes in the DRG and severe, persistent painful symptoms.

Watson CPN et al. reported post-mortem histological findings in a patient with PNH in the last 5 years of life affecting the T7-T8 dermatomes. They found dorsal horn atrophy with loss of myelin and axon at levels T4-T8, but associated cell loss and fibrosis at the T8 ganglion only [22, 23]. When comparing post-mortem histology in similar patients, who suffered long-lasting PNH, to patients that had a resolution of shingles without PNH, the patients without pain had no dorsal horn atrophy. Interestingly, patients with PNH consistently showed structural changes in only one ganglion, despite multiple dermatome levels being involved [22]. In addition, peripheral nerves also undergo similar changes. A significant reduction of small fiber terminals has been demonstrated in skin biopsies [24].

At a molecular level, VZV-mediated nerve injury leads to increased expression of type III Na^+^ channels and upregulation of Ca^2+^ channels [2, 21]. Various A-type voltage-gated K^+^ channels have been identified in the DRG, and a reduction in their activity leads to neuronal hypersensitivity and seems to induce a chronic pain phenotype in animal models [25]. Transient receptor potential channel, subfamily V, member 1 (TRPV1) is another receptor in the dorsal ganglion that, when activated, increases entry of Ca^2+^ leading to oxidative distress and apoptosis. Upregulation of this receptor has been implicated in neuropathic pain models as well [2]. Furthermore, damage to a peripheral nerve can also produce a cascade of inflammatory cells and cytokine release within the DRG that promotes hyperexcitability, thus promoting mechanical allodynia and persistent pain [2].

Clinically, patients may present with pain that is described as spontaneous, burning, aching, or deep. Unbearable itching, paroxysmal pain, and allodynia from mechanical and/or thermal stimuli may also be present. The difference in symptoms and signs among patients correlates with the previously described spectrum of mechanisms [10].

According to Baron and colleagues, the predominant cluster in postherpetic neuralgia is mechanical hyperalgesia [11].

For each of Baron’s subgroups, when topical and systemic drugs do not provide adequate pain relief, invasive treatments should be considered. These invasive treatments include epidural injections, peripheral neuromodulation, DRG neuromodulation, spinal cord stimulation, and DRG stimulation [26]. For example, DRG stimulation has been shown to be particularly beneficial in treating PHN even in cases where SCS has failed [2].

## Methods

All patients underwent DRG radiofrequency as a diagnostic/therapeutic step before considering implantation of a DRG neurostimulator.

**Fig. 2:**
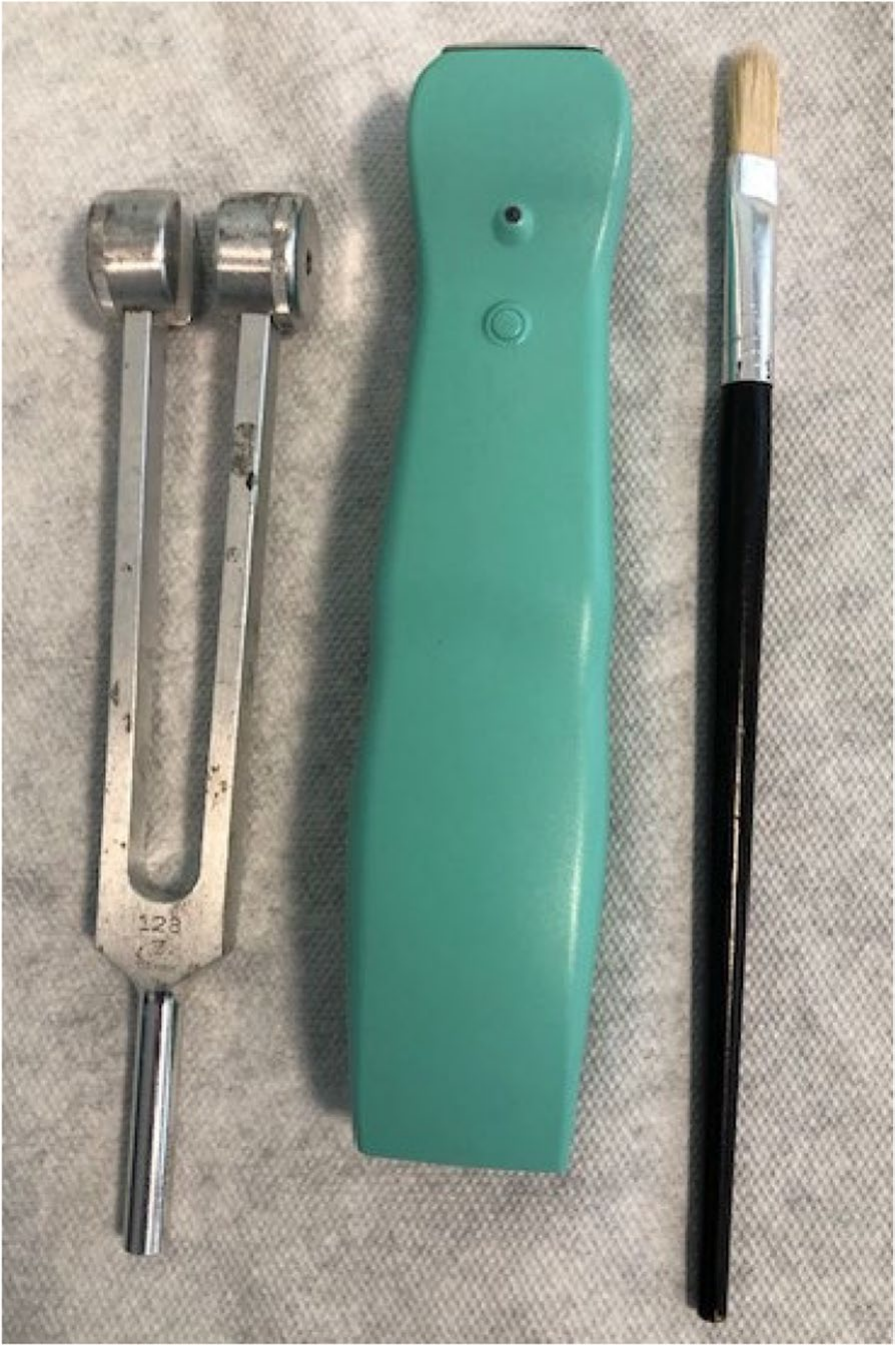
Instruments for bedside evaluation

Patients were evaluated with bedside neurological evaluation for sensory and motor deficits [27]. The instruments to evaluate different fiber integrity included a brush for tactile Aβ fibers, a room temperature (20 °C) metal tuning fork for cold Aδ fibers, and a thermal tester (39 °C) for warm C fibers (Fig. 2).

**Fig. 3:**
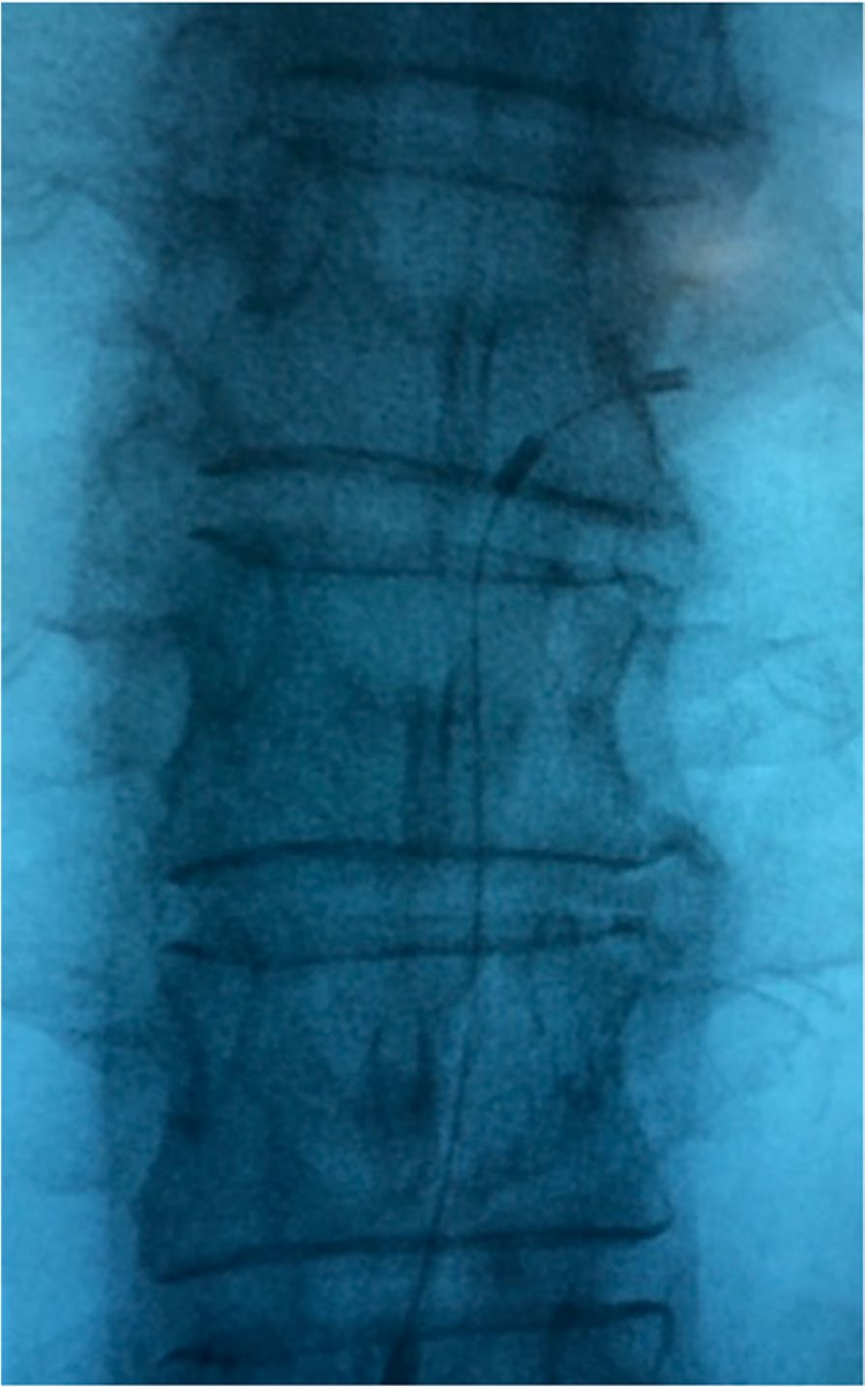
Thoracic lead for pulsed radiofrequency

Using an 8-cm Tuohy needle (18 G) and fluoroscopic guidance, access to the epidural space was obtained, and electrodes were inserted to stimulate various DRG levels in the posterior superior aspect of the foramen. A bipolar lead was introduced via the interlaminar approach with the same method used for DRG lead implant at the thoracic level (Fig. 3) (Easytrode, Bioampere Research, Italy).

**Fig. 4:**
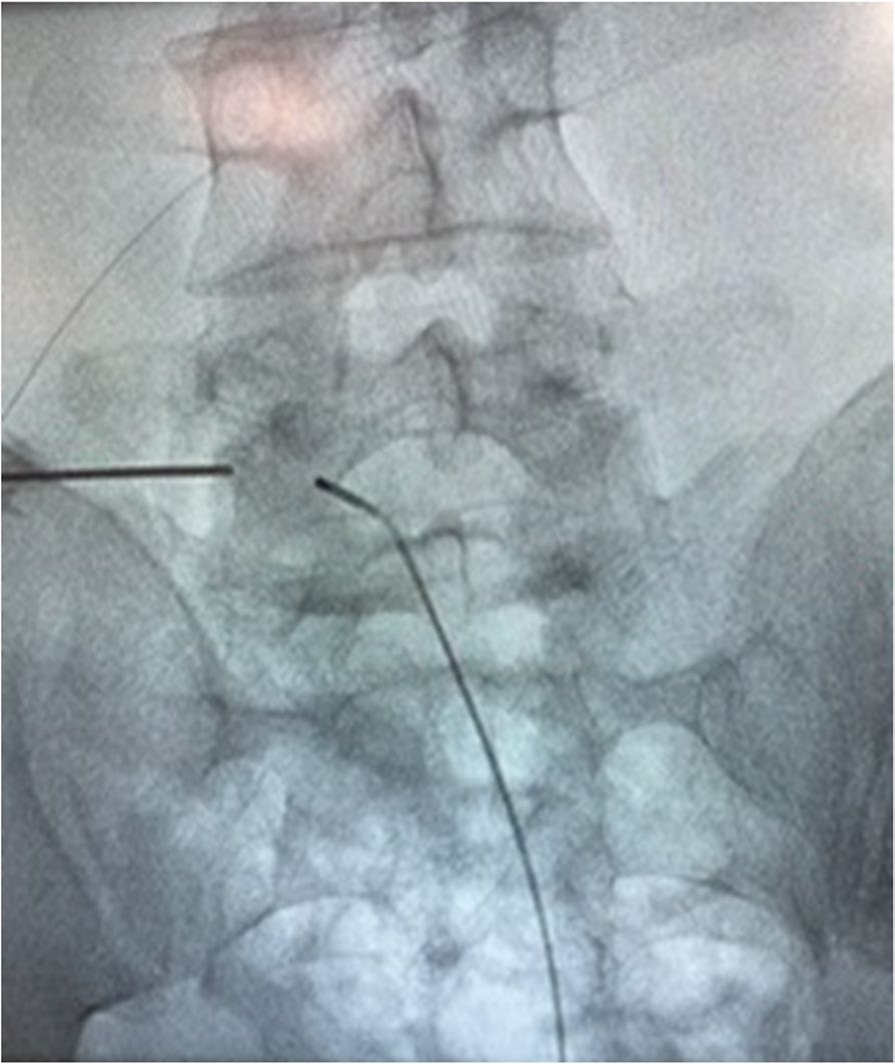
Lumbar lead for pulsed radiofrequency

Access to the epidural space was obtained by inserting the leads caudally, through the sacral hiatus, or laterally, through the intraforaminal space, for bipolar stimulation of lumbar and sacral roots (Fig. 4) (Micro Steer, Acacia, Italy).

Sensory stimulation was applied at 50 Hz and gradually increased from 0.1 V until the patient reported paresthesia or until a maximum intensity of 2 V was reached. Patients were asked to describe where the stimulation was felt and outline the anatomical area the paresthesia covered [9]. The threshold intensity and the percentage of anatomical coverage were then recorded. Next, motor stimulation was applied at 2 Hz until muscle twitching was reported by the patient or observed by the physician.

## Case presentations and results

### Patient 1 case presentation

Fifty-six-year-old male. He presented with continuous pain in the left lower limb, mainly in the foot that was worsened with weight-bearing. The pain started after a stab wound to the back about 20 years ago. He was referred by a colleague that had diagnosed a sciatic nerve/lumbosacral plexus lesion, but the patient’s personal medical records that included neurophysiological and radiological studies could not be found. Previous treatments with opioids, gabapentinoids, and antidepressants were ineffective. Treatment with oxycodone 10 mg/acetaminophen 500 mg, 3 times/day, was ongoing but remained largely ineffective. In addition, the patient was implanted 4 years prior with a high-frequency spinal cord stimulator that was no longer useful.

On inspection, the patient had a scar on the left lower lumbar region. On examination, no significant motor deficit was found. Tactile and thermal anesthesia in the foot and mechanical allodynia in the posterior aspect of the calf were observed.

Clinical evaluation seemed to correspond with Baron’s “sensory loss” model.

### Patient 1 results

RF was tested on the DRGs of L5 and S1 on the left side; paresthesia was not evoked. However, when the stimulation was above 1.5 V, painful muscle contractions in the leg were found. This finding demonstrated a ganglionic or preganglionic lesion involving large-diameter, sensitive fibers without motor fiber involvement. DRG PRF gave no effect, and DRG stimulation was considered not appropriate. The epidural thoracic lead was repositioned with only minimal pain control from high-frequency stimulation.

### Patient 2 case presentation

Patient 2 is a 23-year-old male who had a motorcycle accident 6 months prior with trauma to the left arm, right knee, and left leg. The right knee had paralysis of the external popliteal sciatic nerve and the left leg had a vascular injury that required amputation at the level of the proximal thigh. In addition, the accident caused fracture and dislocation of the left pelvis with injury to the lumbosacral plexus (diagnosed with MRI of the pelvis). The patient presented with episodic, spontaneous pain in the left gluteal region and the posterior-lateral aspect of the stump. MRI of the spine was performed, but no significant spinal damage was reported.

Current treatment included opioids, pregabalin, carbamazepine, clonazepam, and amitriptyline with partial pain relief.

The patient was still on a rehabilitation program to regain the ability to walk.

On clinical evaluation, anesthesia in the inferior gluteal region and stump suggested the “sensory loss” phenotype as suggested by Baron’s clusters.

### Patient 2 results

RF stimulation was tested on left-sided L5 and S1 DRGs. At both levels, no paresthesia was provoked along the nerve root territory, but localized pain was evoked in the lower back and buttocks when pulse intensity was above 1.5 V. This was evidence of a probable ganglionic or preganglionic lesion. It was not possible to advance the lead to a lumbar level higher than L4-L5. A follow-up MRI demonstrated meningoceles occupying the vertebral canal in the T10-S2 segment on the left side and L1-L2 on the right side due to root avulsion. DRG stimulation was not feasible; a spinal cord stimulation trial was proposed but not performed due to patient choice.

### Patient 3 case presentation

Patient 3 is a 91-year-old female with a femoral fracture 5 years ago that led to a total hip replacement. She described continuous, spontaneous pain in the right leg, mainly in the ankle and foot, that did not affect her sleep but limited her daily activity. After surgery, she reported a distal paralysis of the left lower limb with progressive recovery of muscle strength but persistent pain. Neurophysiological evaluation demonstrated a sciatic nerve lesion. She was treated with various injections, physical therapy, antiepileptics, antidepressants, and transdermal fentanyl 62.5 µg/h with minimal benefit.

At clinical evaluation, mechanical allodynia was found in the L5 dermatome, and cold hypoesthesia was present around the L5 and S1 dermatomes. In addition, warm hypoesthesia was found in the same territories. This clinical picture seemed most in line with the characteristics of Baron’s “mechanical hyperalgesia” phenotype.

### Patient 3 results

RF stimulation was tested on right-sided DRGs at the L5 and S1 level; pain was evoked at both levels followed by paresthesia at the intensity of 0.9 V, demonstrating a probable ganglionic or preganglionic lesion of large-diameter fibers. DRG PRF gave no benefit; the patient underwent SCS implantation with partial pain relief.

### Patient 4 case presentation

Patient 4 is an 85-year-old female with diabetes who had continuous and sudden pain, mainly localized to the L5-S1 area of the left leg. She also had motor function impairment with neurological claudication. Pain appeared a year prior to our medical visit and was initially improved by epidural injections that lost efficacy in the last 3 months. Opioids, gabapentinoids, and duloxetine provided no relief.

The clinical exam revealed hypoesthesia to mechanical and cold stimulation in the peripheral area of L5 and S1 of the left leg. There was anesthesia to warm sensations in the same area. Baron’s “sensory loss” phenotype thus seemed most appropriate.

### Patient 4 results

RF stimulation was tested on left-sided DRGs at the L5, S1 level; it was not possible to evoke paresthesia in the territories of the nerve roots. At intensities > 1.5 V, the patient felt paresthesia in the lower lumbar region with leg muscle contractions; thus, a ganglionic or preganglionic lesion involving large and small diameter fibers was hypothesized. Pulsed radiofrequency provided long-lasting pain relief.

### Patient 5 case presentation

Patient 6 is a 68**-**year-old male who had been suffering from PHN in the T1-T3 dermatomes in the left thoracic area and medial aspect of the arm for 20 years. He tried amitriptyline, duloxetine, gabapentinoids, topical lidocaine patches, capsaicin pads, and opioids with poor pain relief and/or side effects. Ongoing therapy included oxycodone 20 mg twice/day, pregabalin 300 mg twice/day, and amitriptyline 10 mg once/day. He described continuous, spontaneous pain that fluctuated in intensity with painful sensations on light touch. Pain often limited daily life activities. On clinical evaluation, there was mechanical allodynia along the dermatomes with mild cold allodynia in the same area except the axillary area where there was cold hypoesthesia. Anesthesia to warm sensations in the painful area was also found. Clinical evaluation seemed in line with Baron’s “mechanical hyperalgesia” model with predominant small fiber loss.

### Patient 5 results

RF was tested at the left T1 level with paresthesia in the ulnar aspect of the hand and forearm but not in the medial arm, axilla, or chest. At the T2 level, pain was felt before paresthesia at 1 V. This pain almost covered the patient’s usual painful area, demonstrating a lesion involving large fibers more than small fibers. Upon T3 stimulation at 0.4 V, paresthesia was appreciated in the more caudal part of the painful area. The injection of 0.5 ml of 0.5% lidocaine on the T2 ganglion made the pain disappear for about 24 h. The patient had no pain relief with DRG PRF, but two DRG leads were implanted in T2 and T3 with 40% pain relief which, for the patient, was a good result (Fig. 1).

### Patient 6 case presentation

Patient 6 is an 82-year-old male who had been suffering from postherpetic neuralgia in the T5-T7 dermatomes of his right thorax for the last 18 months. He had tried gabapentinoids, carbamazepine, tramadol, and opioids with little to no benefit; he was still in treatment with gabapentin 100 mg, six times/day, acetaminophen 1000 twice/day, and lidocaine 5%, 1 patch in the evening. He underwent implantation of a peripheral nerve stimulator with temporary pain relief 6 months ago, but this relief had since subsided.

The patient described continuous pain along the affected dermatomes that interfered with his sleep quality.

On clinical evaluation, mechanical allodynia was found in the antero-lateral territory of T5 and T6 nerve roots. In addition, thermal allodynia to cold and warm stimulus was found in the lateral territory of the same roots.

Thermal hypoesthesia was also observed in the anterior territory. Clinical evaluation suggested the “thermal hyperalgesia” model.

## Patient 6 results

RF was tested on T5 on the right side with paresthesia covering the painful area only at an intensity above 2 V. No pain was felt upon T5 stimulation, demonstrating a lesion involving both large and small diameter fibers. T6 paresthesia was evoked in the painful area at 0.5 V. The injection of 0.5 ml of 0.5% lidocaine at T5 and T6 DRG did not improve pain intensity significantly. The patient felt no effect from DGR PRF; a DRG lead was implanted in T6 with improvement of allodynia but no pain relief.

**Fig. 5:**
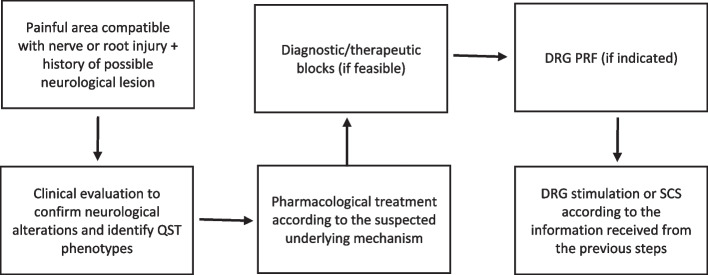
Proposed diagnostic and treatment flowchart. QST quantitative sensory testing, DRG dorsal root ganglia, PRF pulsed radiofrequency, SCS spinal cord stimulation

Table 1 provides a summary of these findings, and Fig. 5 provides a proposed diagnostic and treatment algorithm using PRF of the DRG.

**Table 1:** Table of clinical comparison between the 6 cases

	Patient 1	Patient 2	Patient 3	Patient 4	Patient 5	Patient 6
Clinical history	Lower limb pain after stabbing	Motorcycle accident leading to left leg amputation with stump and left gluteal pain	Continuous right ankle and foot pain after total hip replacement	Elderly diabetic with constant lower leg pain	PNH in the T1-T3 dermatomes	PNH in the T5-T7 dermatomes
Clinical findings	Tactile and thermal anesthesia in the foot and mechanical allodynia in the calf	Anesthesia in the inferior gluteal region and stump	Mechanical allodynia in the L5 dermatome and cold hypoesthesia around the L5 and S1 dermatomes	Hypoesthesia to mechanical and cold stimulation in the peripheral area of L5 and S1 of the left leg	Pain with light touch, anesthesia to heat, and cold + mechanical allodynia along the T1-T3 area	Mechanical allodynia at the T5 and T6 levels with thermal allodynia
Baron’s proposed mechanism	Sensory loss	Sensory loss	Mechanical hyperalgesia	Sensory loss	Mechanical hyperalgesia	Thermal hyperalgesia
DRG PRF results	RF at L5 and S1 showed no paresthesia at lower voltage but muscle contraction at higher voltage	RF at L5 and S1 showed no paresthesia at lower voltage but pain in the lower back and buttocks at higher voltage	RF at L5 and S1 evoked pain and paresthesia	RF at L5 and S1 showed paresthesia at lower voltage and paresthesia with muscle contraction at higher voltage	RF at T1-T3 covered the painful area with paresthesia	RF at T5 created paresthesia only at high voltage
Probable mechanism	Ganglionic or preganglionic lesion of large-diameter fibers	Ganglionic or preganglionic lesion	Ganglionic or preganglionic lesion of large-diameter fibers	Ganglionic or preganglionic lesion involving large and small diameter fibers	PNH lesion involving large fibers more than small fibers	PNH lesion involving many large and small fibers
Treatment	Repositioned spinal cord stimulator lead	Spinal cord stimulator trial was proposed but not performed	Spinal cord stimulator	Pulsed radiofrequency	Ganglionic stimulator lead placement	DRG lead placement with improvement of allodynia only

*RF* radiofrequency, *PNH* postherpetic neuralgia

## Discussion

In patients with neuropathic pain due to lesions or diseases involving spinal roots, it is sometimes difficult to define the exact site and extent of the lesion with clinical and instrumental tools alone. Understanding the site, extent, and mechanisms underlying neuropathic pain syndromes improves the outcomes of targeted, invasive therapies [12].

The DRG is a critical structure in pain pathophysiology when directly involved in the lesion as well as when the lesion is peripheral [28]. Furthermore, the integrity of the DRG and its central axons is crucial for differentiating peripheral neuropathic pain from deafferentation pain; in the first case, the DRG can be an important target [29], while in the second one, the target should be second-order neurons. The observations we have made in these patients demonstrate that radiofrequency stimulation of the DRG can give us useful information on the integrity and functionality of the ganglion.

### Radiculopathy/plexus lesions

Herniated disks and spinal/foraminal stenosis cause damage to spinal roots. This damage can be at the preganglionic, ganglionic, or postganglionic level. Radiology can help define the site of the lesion and targeted neurophysiological examinations can help define the level of the lesion. However, sometimes the lesion can involve the root at different levels, and neurophysiology cannot give precise information. In traumatic or post-surgical injuries, different lesions could coexist. This is the case for our stab wound, pelvic trauma, and hip fracture patients.

In patient 1, it was not possible to demonstrate the exact site and depth of the lesion, but it was hypothesized to be a lesion of the emerging roots in the spinal canal at the preganglionic level.

In patient 2, MRI demonstrated a lumbosacral plexus lesion and neurophysiological testing was impossible; DRG PRF stimulation demonstrated a preganglionic or ganglionic lesion. This finding was confirmed with MRI demonstrating meningoceles due to root avulsion with deafferentation pain.

In patient 3, a traumatic sciatic nerve lesion was hypothesized, but RF demonstrated a preganglionic lesion probably due to local anesthetic toxicity during spinal anesthesia in a patient with spinal stenosis.

### Postherpetic neuralgia

In patients with postherpetic neuralgia, we could define the DRG involved by the disease and type of fibers affected with more precision than clinical evaluation.

Patient 5 seemed to be classified by the phenotype of “mechanical hyperalgesia” based on Baron’s clusters, and thus involvement of mostly small fibers was expected. Stimulation of the DRG however helped us document the predominant loss of large fibers. The affected DRG could be targeted by neurostimulation with some benefit in terms of pain relief.

Patient 6 was classified under the phenotype “thermal hyperalgesia” according to Baron, with the hypothesis of hyperactivity of surviving nociceptors. We, however, observed a lesion involving all types of DRG fibers with positive symptoms that could be due to the overlapping of fibers from adjacent DRGs while spontaneous pain could be attributed to deafferentation of second-order neurons. In this case, DRG stimulation was effective only on the allodynia but not on the spontaneous pain.

When considering DRG stimulation as a therapeutic option, it is important to know whether the DRG or the neighboring ganglia are functional. When considering a SCS trial, it is fundamental to know if central axons of large-diameter fibers are still alive and whether they can be targeted in the dorsal columns to “close the gate” [30]. If pain arises from ectopic activity of deafferented second-order neurons, then perhaps waveforms that target the dorsal horns should be chosen [31]. Clinical evaluation can give us important information and Baron’s proposed mechanisms can help classify that information to better understand the underlying pathology and create a treatment plan. For example, implicit in the “sensory loss” phenotype is the idea that the site of the lesion cannot always be identified; therefore, PRF of the DRG can help narrow down the diagnosis. Within this mechanistic phenotype, if the lesion involves the ganglion and no or minor activity of the first-order neuron seems present through PRF of the DRG, then pain likely arises from the second-order neuron. DRG stimulation is therefore not a feasible treatment option (though stimulation of the adjacent DRG could be considered), and SCS is also less likely to be effective.

When evaluating patients, especially at the thoracic level where dermatomes are narrower, it must be remembered that dermatomes can have inconsistencies and that nerve roots territories can overlap [32]. This phenomenon could lead someone who is following Baron’s mechanisms to conclude that the pain is categorized under the “mechanical hyperalgesia” phenotype while DRG stimulation demonstrates “sensory loss” at one DRG level.

Therefore, whether considering stimulation of the ganglia or stimulation of the spinal cord, PRF of the DRG can provide valuable diagnostic information.

One limitation of the study is that the patients were not studied with a complete Quantitative Sensory Testing but only with bedside evaluation intended to evaluate the functionality of Aβ, Aδ, and C fibers. In addition, despite the potential advantages demonstrated in the literature, stimulation of the DRG may only provide therapeutic benefits for a few months, and the mechanism of action is not yet completely understood [1]*.*

## Conclusions

Dorsal root ganglion pulsed radiofrequency is a therapeutic option that, in the case of peripheral nerve or nerve root lesions, should be considered in the therapeutic algorithm before considering more invasive techniques. RF stimulation has been shown to help verify the correct position of the lead (or needle) and map the target ganglia before inserting a DRG stimulation device [8, 9]. Research on the benefits of PRF of the DRG in localizing and treating radicular pain is lacking. This study adds to the literature by showing that RF stimulation can help us understand the functionality of the ganglia and the understanding of pain mechanisms in difficult cases of neuropathic pain.

## Data Availability

The study was observational, retrospective. The patients were treated according to the Unit protocols. The patients were included because we noted unexpected information from DRG stimulation. The data are recorded in patient files.

## Abbreviations

DMA: Dynamic mechanical allodynia
DRG: Dorsal root ganglia
MRI: Magnetic resonance imaging
PHN: Postherpetic neuralgia
PRF: Pulsed radiofrequency
QST: Quantitative sensory testing
SCS: Spinal cord stimulation
VZV: Varicella-Zoster virus

## Data Availability

No datasets were generated or analysed during the current study.
